# Survival of *Lacticaseibacillus rhamnosus* LRB in a Potential Synbiotic Oat Beverage: Insights into Cell-Matrix Interactions During *In Vitro* Digestion

**DOI:** 10.1007/s11130-026-01550-9

**Published:** 2026-07-22

**Authors:** Giovanna Alexandre Fabiano, Douglas Xavier-Santos, Stefhani Andrioli Romero, Rodrigo Stein Pizani, Paulo César Martins Alves, Marcos Tadeu Nolasco da Silva, Mauricio Ariel Rostagno, Adriane Elisabete Costa Antunes

**Affiliations:** 1 School of Applied Sciences, Universidade Estadual de Campinas (UNICAMP), Limeira, SP Brazil; 2 Department of Food Science and Nutrition, Faculty of Food Engineering, Universidade Estadual de Campinas (UNICAMP), Campinas, SP Brazil; 3 Center for Investigation in Pediatrics, School of Medical Sciences, Universidade Estadual de Campinas (UNICAMP), Campinas, SP Brazil

**Keywords:** Plant-based, Flow cytometry, Probiotics, Prebiotics, Gastrointestinal stress, Functional foods

## Abstract

The growing demand for plant-based functional foods has positioned oats as a promising matrix for developing synbiotic products, given their high fiber content. This study aimed to develop a fermented oat beverage supplemented with inulin to evaluate the survival and morphological integrity of *Lacticaseibacillus rhamnosus* LRB during *in vitro* gastrointestinal digestion. A bovine milk beverage with the same added ingredients served as a reference matrix (control). The oat beverage reached the target pH (~ 4.5) faster than the bovine milk beverage and maintained the stability of the *L. rhamnosus* LRB during 28 days of storage (8.34 ± 0.02 log cfu.mL^− 1^; ∆ log = -0.27). After simulated in vitro digestion, the oat matrix ensured high recovery rates (> 7 log cfu.mL^− 1^). Flow cytometry revealed a significantly lower percentage of dead cells in the oat beverage (2.10%) during the gastric digestion phase. Scanning electron microscopy confirmed the structural stress induced by digestion in the lactic acid culture. Additionally, the oat beverage maintained substantial antioxidant activity in the intestinal digested fraction. These results demonstrate that the oat-inulin matrix is a technologically viable vehicle for *L. rhamnosus* LRB, providing more than 10 log cfu per serving and meeting the criteria for potential probiotic food. This study elucidates the protective role of the oat matrix and supports the development of high-performance plant-based functional beverages.

**Supplementary Information:**

The online version contains supplementary material available at https://doi.org/10.1007/s11130-026-01550-9.

## Introduction

The growing demand for functional foods has driven the development of synbiotic beverages. These beverages combine probiotics and prebiotics to modulate the gut microbiota and support intestinal homeostasis [1]. Dairy matrices have traditionally dominated this market, as their protein and lipid composition protect microorganisms during processing, storage, and gastrointestinal transit [2]. However, rising concerns like lactose intolerance, milk protein allergies, and sustainability have propelled the search for plant-based alternatives. In this context, oats (*Avena sativa* L.) emerge as a promising vehicle due to their high digestibility and unique composition, rich in soluble fibers, particularly *β*-glucans [3]. To enhance synbiotic potential, isolated prebiotics, such as inulin—a linear fructan resistant to human digestive enzymes—are often added to improve health benefits and texture [4].

The efficacy of these functional products depends on maintaining the viability of the incorporated microorganisms throughout shelf-life and digestion. *Lacticaseibacillus rhamnosus* (previously classified as *Lactobacillus rhamnosus*) is widely used due to its facultative heterofermentative nature and the probiotic properties of some strains [5]. Specifically, *Lacticaseibacillus rhamnosus* LRB is a novel candidate. It was isolated from a healthy human milk tooth and is genetically similar to the well-characterized and safe strain *L. rhamnosus* GG (ATCC 53103) [6]. Although the LRB strain has recently demonstrated excellent performance and stability in a dairy matrix, its interaction with non-dairy plant matrices and its structural survival during gastrointestinal transit remain completely unknown [7].

Traditionally, culture viability is monitored by counting colony-forming units (cfu). This method cannot detect cells in a viable but non-culturable (VBNC) state [8]. To overcome these limitations, flow cytometry (FC) and scanning electron microscopy (SEM) emerge as essential tools. They can assess cytoplasmic membrane integrity and/or investigate bacterial adhesion to the matrix, which can act as a physical shield [9]. Therefore, this study aims to develop a novel, potentially synbiotic, fermented oat beverage supplemented with inulin to ensure the survival and cellular morphology of *Lacticaseibacillus rhamnosus* LRB after in vitro digestion. This work seeks to clarify the interaction between the plant matrix and the lactic acid culture using advanced analytical tools, providing a scientific basis for high-performance plant-based products.

## Materials and Methods

### Ingredients and Microorganism

Rolled oats (PepsiCo, Brazil), chicory inulin (DP > 10, Orafti GR, Beneo, Belgium), skimmed milk powder (Piracanjuba, Brazil), and sucrose (União, Brazil) were purchased locally in Limeira (São Paulo, Brazil). The commercial culture *L. rhamnosus* LRB was supplied by Sacco System (Cadorago, Italy).

### Preparation and Fermentation of Beverages

The oat beverage base was prepared by processing rolled oats with filtered water (1:20, w/v), supplemented with 3% (w/v) inulin and 7% (w/v) sucrose using a Thermomix (TM6-1, Vorwerk, Germany), filtered through a 150-mesh nylon membrane (106 μm), and autoclaved (Phoenix Luferco, Brazil) at 115 °C for 18 min. A reference milk beverage base (control) was prepared with skimmed milk powder (1:10 w/v) under identical sucrose, inulin, and processing conditions.

For inoculation, 0.1 g of freeze-dried *L. rhamnosus* LRB was activated in 50 mL of MRS broth (Merck KGaA, Germany) at 37 °C for 24 h, then centrifuged (Solab, Brazil) at 2,660 × *g* for 5 min at room temperature, washed three times with 0.9% sterile saline, and inoculated into both beverage bases (~ 10 log cfu.mL^− 1^). Static fermentation at 37 °C (BOD, Tecnal, Brazil) in sterile 500 mL glass bottles with screw caps proceeded until the oat beverage reached a pH of 4.5 ± 0.1, defining the standardized fermentation time applied to both matrices. pH (digital pH meter; MS Tecnopon, Brazil) and titratable acidity (TA, % lactic acid by titration with 0.1 N NaOH) were monitored. Details and mathematical equations for TA are available in the Supplementary Material (Eq. S1). The fermented oat beverage and fermented milk beverage were designated as FOB and FMB, respectively.

### Simulated Gastrointestinal Digestion

The INFOGEST standardized protocol was employed to simulate the oral, gastric, and intestinal phases sequentially [10]. Briefly, 5 mL of each beverage was mixed (1:1, v/v) with 5 mL of simulated salivary fluid containing *ɑ*-amylase (75 U.mL^− 1^; pH 7.0) for 2 min. For the gastric phase, the resulting 10 mL of each bolus was diluted (1:1, v/v) with 10 mL of simulated gastric fluid containing pepsin (2,000 U.mL^− 1^), adjusted to pH 3.0 with 1 M HCl, and incubated for 2 h to obtain gastric chyme (FOB-G and FMB-G). Finally, 20 mL of each gastric chyme was mixed (1:1, v/v) with 20 mL of simulated intestinal fluid containing pancreatin (100 U.mL^− 1^) and bile salts (10 mM), adjusted to pH 7.0 with 1 M NaOH, and incubated for 2 h to obtain the final intestinal fractions (FOB-I and FMB-I). All phases were performed at 37 °C under constant orbital shaking (150 rpm; Tecnal, Brazil). Aliquots were collected at the end of each stage for subsequent analyses.

### Characterization of the Fermented Oat Beverage

The proximate composition (moisture, ash, protein, lipids, and total carbohydrates) and microbiological safety (*Salmonella* spp., *Bacillus cereus*, Enterobacteriaceae, and molds and yeasts) of the newly developed FOB were determined according to official methodologies, as detailed in the Supplementary Material (including Eq. S2 to S7). Antioxidant activity was evaluated in FOB, FOB-G, and FOB-I. Briefly, samples were centrifuged (10,000 × *g*, 10 min, 4 °C) to obtain the supernatants used for the analysis. The DPPH (517 nm), ABTS (730 nm), and FRAP (596 nm) spectrophotometric assays were performed using microplate formats. The complete experimental protocols, reagent preparation, and calibration curves for these assays are detailed in the Supplementary Material.

#### Storage stability and viability of *L. rhamnosus* LRB

Viable cell counts of *L. rhamnosus* LRB in FOB and FMB were monitored every seven days during a 28-day refrigerated storage period (5–10 °C) using the pour-plate method on MRS agar (Merck, Germany) at 37 °C for 72 h. Results were expressed as log cfu.mL^− 1^. Logarithmic variation (∆ log) was calculated using Equation S8 (Supplementary Material). Plate counts before and after simulated digestion were complemented by flow cytometry (FC) to compare the protective effect of FOB and FMB. The initial sample counts were normalized using phosphate-buffered saline (PBS, pH 7.0; Laborclin, Brazil). Then, 100 µL were incubated with 10 µL of SYTO-9 (5 mmol.L^− 1^; Thermo Fisher Scientific, USA) and 1 µL of PI (1 mmol.L^− 1^; Sigma-Aldrich, Brazil) to differentiate viable from damaged cells [11]. After incubation, 1,000 µL of PBS was added, and the solutions were centrifuged (2,300 × *g*, 4 °C) for 10 min. The supernatants were discarded, and the pellets were resuspended in 300 µL of PBS and analyzed using a FACSVerse flow cytometer (BD Biosciences, USA). Unstained and autoclaved dead cells were used as controls, and uninoculated matrix backgrounds were subtracted.

### Scanning Electron Microscopy (SEM)

Bacterial morphology and cell-matrix interactions before and after digestion were observed via SEM in both beverages [12]. Samples were filtered through polyether sulfone membranes (0.22 μm, Merck Millipore, Ireland), fixed with 2.5% (v/v) glutaraldehyde in 0.2 M phosphate buffer for 24 h, and dehydrated using a graded ethanol series (25–100%). After CO_2_ critical point drying (Leica EM CPD 300) and gold-sputtering (Balzers SCD-050 Sputter Coater), the images were captured using FEG TESCAN MIRA 4 microscope (SEM-FEG-TESCAN, Czech Republic) at 15 keV and magnifications ranging from 10,000 to 15,000x.

### Statistical Analysis

Assays were performed in independent biological and technical triplicates. Data normality and homoscedasticity were verified using the Shapiro-Wilk and Levene/Bartlett tests, respectively. Student’s *t*-test or repeated-measures ANOVA followed by Tukey’s *post-hoc* test were applied for parametric data, while Friedman and Dunn’s tests were used for nonparametric datasets (GraphPad Prism version 8.0.2; San Diego, USA), adopting *p* < 0.05.

## Results and Discussion

### Fermentation Parameters

Regarding fermentation parameters (Table 1), the FOB reached the target pH of 4.5 ± 0.1 within 3 h, while FMB required 12 h to achieve the same pH level under identical conditions with high counts of *L. rhamnosus* LRB (~ 10 log cfu.mL^− 1^). To ensure methodological consistency and enable comparison, the fermentation time was standardized to 4 h for both substrates, using the FOB as the primary reference. Although the milk matrix had not reached pH 4.5, this standardization was a strategic decision. Since the main objective of this study was to develop a functional plant-based product—and not a traditional dairy beverage—bovine milk was used strictly as a reference control to evaluate the delivery efficiency of the *L. rhamnosus* LRB.

**Table 1:** Acidification parameters (pH and lactic acid content) of oat beverage (FOB) and milk beverage (FMB) fermented by *L. rhamnosus* LRB

Fermentation time (hours)	FOB		FMB	
	pH	Lactic acid (%)	pH	Lactic acid (%)
0	6.59 ± 0.04^Aa^	0.04 ± 0.00^Ab^	6.68 ± 0.07^Aa^	0.27 ± 0.04^Aa^
1	5.65 ± 0.09^Bb^	0.05 ± 0.01^Ab^	6.58 ± 0.10^Aa^	0.34 ± 0.04^ABa^
2	5.08 ± 0.25^Bb^	0.06 ± 0.01^ABb^	6.45 ± 0.18^Aa^	0.38 ± 0.03^Ba^
3	4.50 ± 0.15^Cb^	0.08 ± 0.01^Bb^	6.31 ± 0.32^Aa^	0.43 ± 0.03^Ca^
4	4.20 ± 0.28^Cb^	0.09 ± 0.01^Bb^	6.09 ± 0.30^Aa^	0.48 ± 0.02^Ca^

Data are expressed as mean ± standard deviation. Capital letters within the same column indicate significant differences over fermentation time within the same matrix according to Tukey’s multiple comparison test. Lowercase letters within the same row indicate significant differences between matrices at the same fermentation time, as determined by Sidak’s multiple comparisons test (*p* < 0.05)

This approach reveals that the oat matrix was notably more responsive to fermentation by *L. rhamnosus* LRB. Although a 4-hour fermentation period is typically sufficient for dairy beverages using standard starter cultures, this duration was insufficient for the *L. rhamnosus* LRB monoculture in FMB to reach the desired acidity.

According to Table 1, a sharp decline in pH was observed in the FOB, whereas a subtle decrease occurred in the FMB (*p* < 0.05), accompanied by distinct lactic acid production depending on the food matrix, which reached 0.09% ± 0.0 in the FOB and 0.48% ± 0.02 in the FMB. This divergence highlights the high buffering capacity of bovine milk, which is widely attributed to its casein-mineral equilibria. Within the mineralized micellar structure, protons generated by lactic acid production shift phosphate speciation and induce the progressive dissolution of micellar calcium phosphate (MCP) into the soluble phase [13, 14]. This pH-responsive mineral redistribution and proton-driven phosphate equilibrium effectively sequester free H^+^ ions, thereby delaying the pH decline despite the higher total organic acid accumulation. In contrast, the lower concentration of buffering components in the oat matrix enabled even a modest lactic acid production to abruptly reduce the pH, confirming the technological advantage in processing time. Furthermore, supplementation with sucrose and inulin provided readily fermentable carbohydrates for *L. rhamnosus* LRB, significantly accelerating organic acid production compared to longer oat fermentations reported in the literature [15, 16].

### Characterization of Fermented Oat Beverage

The proximate composition of the FOB is available in Supplementary Material Table S1, showing a carbohydrate-rich profile (9.12% ± 0.44) enhanced by sucrose and inulin supplementation. The protein and lipid profiles remained consistent with those reported for other oat-based beverages, which are characteristically lower in protein than bovine milk [17, 18]. Furthermore, microbiological safety criteria were fully met after fermentation and throughout the 28 days of refrigerated storage, with no *Salmonella* ssp. detected, and counts < 10 cfu.mL^− 1^ for *B. cereus*, Enterobacteriaceae, and molds and yeasts (Table S2). These results confirmed that the fermentation process and refrigerated storage were effective in maintaining food safety, in accordance with the current Brazilian Normative Instruction No. 161 of 2022 from the National Health Surveillance Agency [19].

Regarding antioxidant activity, Table 2 presents the FOB results from simulated in vitro digestion (FOB-G and FOB-I). A significant reduction in antioxidant activity (*p* < 0.05) was observed across all evaluated methods after the complete digestion. This marked decrease, particularly pronounced during the transition from the gastric to intestinal phase in ABTS and FRAP assays, may be associated with the transition to an alkaline pH in the intestinal stage, potentially promoting the degradation or structural alteration of phenolic compounds. Although the fermentation process is documented to enhance initial antioxidant profiles by releasing bioactive peptides and phenolic compounds, exposure to sequential digestive fluids imposes strict limitations on their stability [1]. Consequently, the residual antioxidant activity detected in intestinal chyme may contribute to the functional properties of the oat beverage in the gut. Furthermore, it is important to emphasize that although this residual antioxidant activity remains relevant, the qualitative and quantitative profiles of specific polyphenols were not characterized in this study, thereby limiting the precise molecular elucidation of the compounds responsible for this bioactivity.

**Table 2:** Antioxidant activity (µmol TE.mL^− 1^) of fermented oat beverage (FOB) before and during simulated in vitro digestion

	DPPH	ABTS	FRAP
FOB	3025.04 ± 105.84^a^	11845.02 ± 64.58^a^	715.27 ± 9.21^a^
FOB-G	2435.17 ± 99.23^a^	8937.15 ± 98.21^b^	252.66 ± 12.60^b^
FOB-I	297.69 ± 6.62^b^	7002.02 ± 119.87^c^	141.11 ± 6.68^c^

Data are expressed as mean ± standard deviation. FOB, fermented oat beverage; FOB-G, fermented oat beverage subjected to simulated gastric digestion; FOB-I, fermented oat beverage subjected to simulated intestinal digestion. Lowercase letters within the same column indicate a significant difference according to Tukey’s multiple comparisons test (*p* < 0.05)

### Survival of the *L. rhamnosus* LRB During Storage and Simulated *In Vitro *Digestion

Table 3 presents the *L. rhamnosus* LRB count and its respective logarithmic variations during 28 days of refrigerated storage. The FOB demonstrated a remarkable protective effect on *L. rhamnosus* LRB, with slightly decreased (∆ log = -0.27), sustaining counts > 8 log cfu.mL^− 1^. Conversely, the FMB exhibited a more pronounced reduction (∆ log = -1.63) by the end of the 28-day period. This suggests that oat components, such as *β*-glucans, may act as protective agents against cold stress for cell membranes. $\:\beta\:$-glucan and inulin are well-established prebiotics, characterized by their selective utilization by beneficial microorganisms and resistance to host digestion. Consequently, studies indicate that these fibers can enhance the viability, growth, and survival of bacteria such as *L. rhamnosus* [20, 21]. Furthermore, inulin and $\:\beta\:$-glucan can form cross-linked hydrogels that serve as protective microenvironments, acting as physical barriers that shield bacterial cells from storage and gastrointestinal stresses [22, 23]. The rapid initial pH decline observed in the FOB may have also triggered acid-stress adaptation mechanisms. This physiological response typically induces the expression of general stress proteins, such as the GroEL and DnaK chaperones, and activates proton-translocating H^+^-ATPases to maintain cytoplasmic pH homeostasis [24, 25]. Although the exact ATPase response depends on the specific strain and stress context, this prior adaptation likely explains why the culture in the FOB exhibited a lower cell death rate when subsequently challenged by cold storage and gastric conditions, compared to the FMB, where the pH decline was slower and delayed.

**Table 3:** Counts (log cfu.mL^− 1^) and logarithmic variation (∆ log) of *L. rhamnosus* LRB in fermented oat and milk beverages (FOB and FMB) during refrigerated storage

Storage time (days)	FOB		FMB	
	*L. rhamnosus* LRB	∆ log	*L. rhamnosus* LRB	∆ log
0	8.61 ± 0.02^Ab^	0.00	9.71 ± 0.02^Aa^	0.00
7	8.50 ± 0.04^ABb^	-0.13	9.32 ± 0.05^Ba^	-0.39
14	8.39 ± 0.00^Bb^	-0.22	9.15 ± 0.02^Ca^	-0.56
21	8.37 ± 0.02^Bb^	-0.24	9.10 ± 0.01^Ca^	-0.61
28	8.34 ± 0.02^Ba^	-0.27	8.08 ± 0.10^Db^	-1.63

Data are expressed as mean ± standard deviation. FOB, fermented oat beverage; FMB, fermented milk beverage. Capital letters within the same column indicate significant differences over time according to Tukey’s multiple comparisons test (*p* < 0.05). Lowercase letters within the same row indicate significant differences between the different food matrices, as determined by Šídák’s multiple comparisons test (*p* < 0.05)

Plant-based beverages contain polyphenols with antimicrobial effects that can impact bacterial viability [26]. However, this viability varies considerably depending on the matrix type, specific strains, and processing or storage conditions. When utilizing oats as a substrate, previous studies have reported that lactic acid bacteria viability reaches approximately 7 to 9 log cfu.mL^− 1^, which corroborates the findings of this study [15, 16]. Furthermore, the beverage developed in the present work exhibited final counts exceeding 10 log cfu *per* 100 mL serving, surpassing the consensus minimum threshold (8 log cfu *per* serving) required for classification as a potential probiotic [26].

**Fig. 1:**
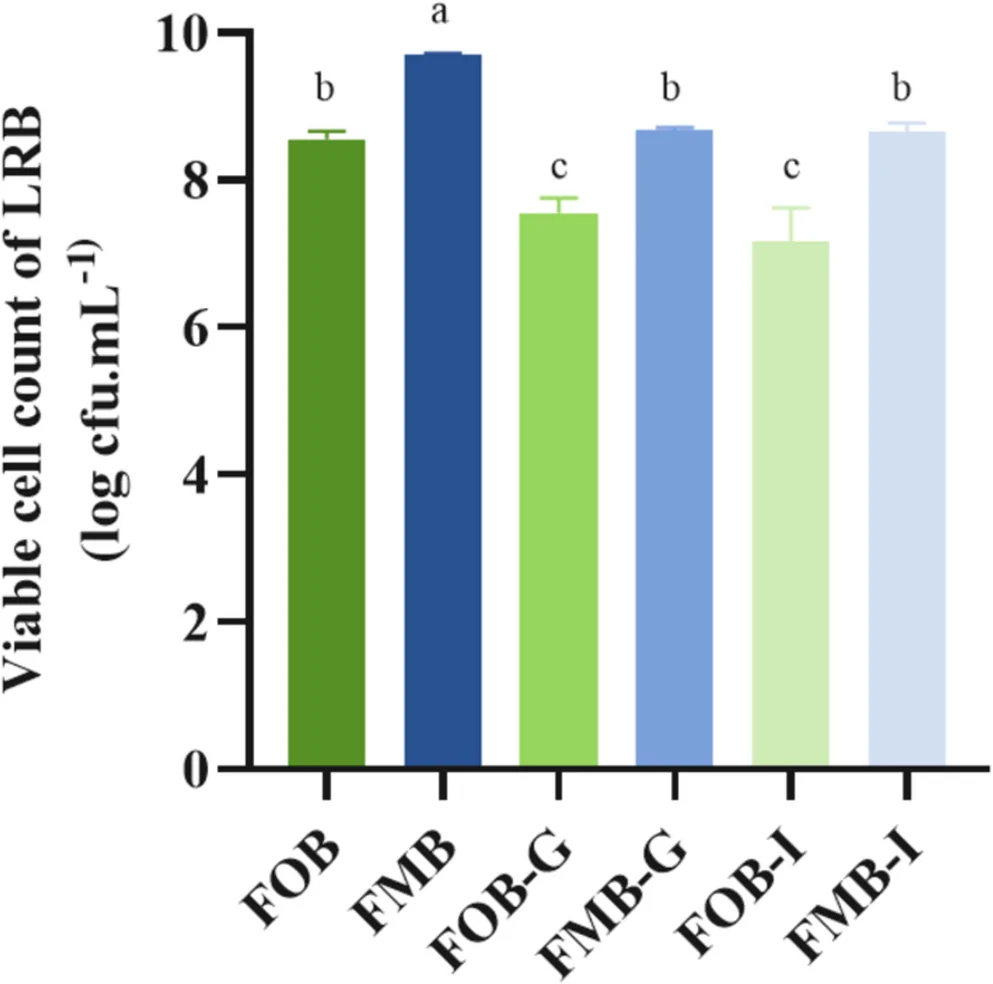
Viable cell counts of *L. rhamnosus* LRB (log cfu.mL− 1) in fermented oat beverage (FOB) and fermented milk beverage (FMB) before and during simulated in vitro digestion. FOB and FMB correspond to samples immediately after fermentation; FOB-G and FMB-G to the gastric phase; and FOB-I and FMB-I to the intestinal phase. Different letters indicate significant differences among all treatment combinations across both beverages and digestion stages simultaneously, as determined by Tukey’s multiple comparisons test (*p* < 0.05)

Regarding simulated *in vitro* digestion in Fig. 1, plate counts revealed that viable cells in the FOB decreased by approximately 1.01 log cycles in the gastric phase and 1.40 log cycles in the intestinal phase, maintaining high final counts (7.16 ± 0.46 log cfu.mL^− 1^). Although the FMB retained slightly higher absolute counts (8.65 ± 0.11 log cfu.mL^− 1^), no statistically significant differences were detected between the gastric and intestinal digestion stages for either matrix, indicating that digestion affected the survival of the lactic culture similarly in both substrates.

**Fig. 2:**
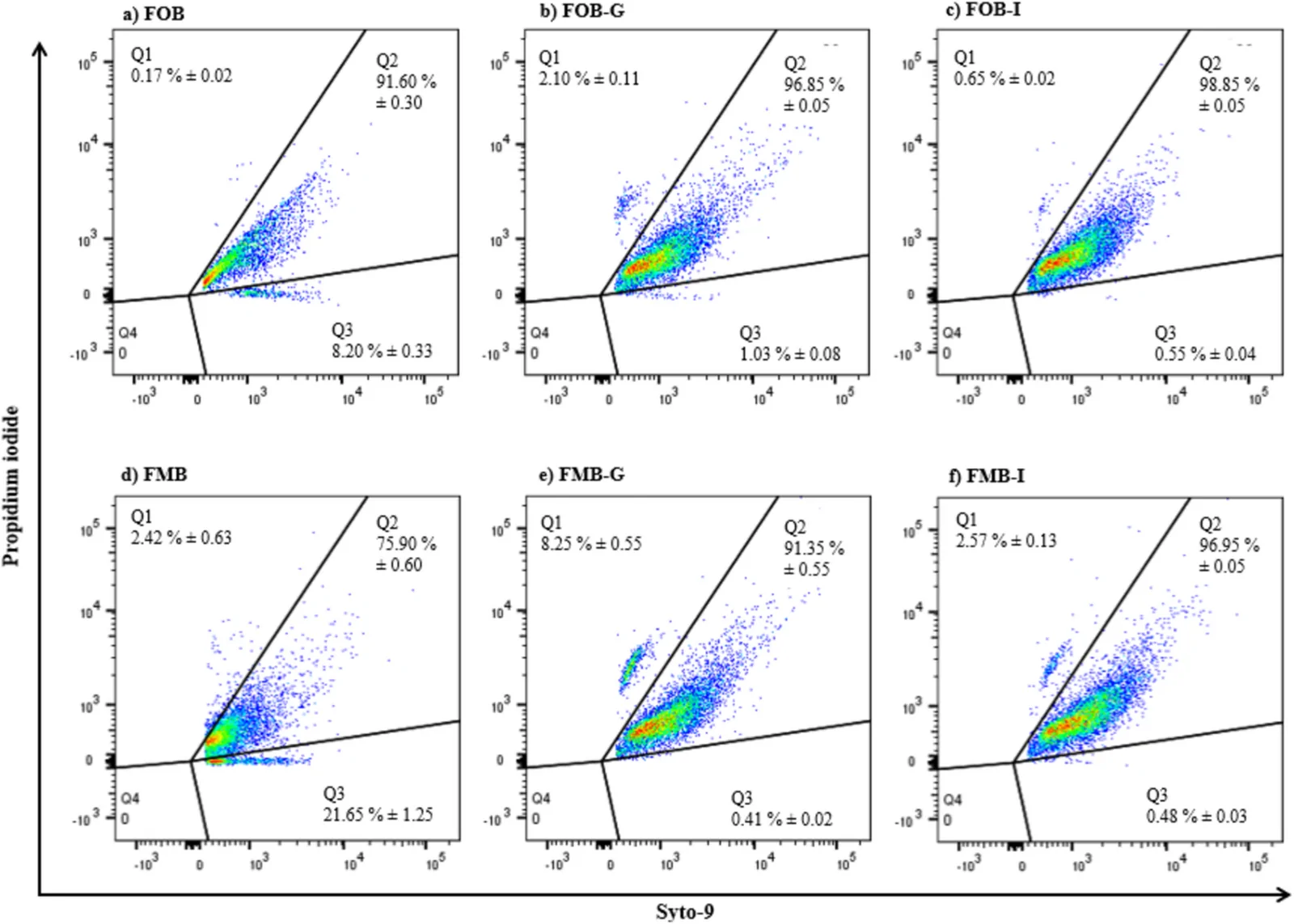
Flow cytometry dot plots of *L. rhamnosus* LRB in fermented oat beverage (FOB) and fermented milk beverage (FMB) before and during simulated in vitro digestion. Panels (**a**,** b**,** c**) correspond to FOB and (**d**,** e**,** f**) to FMB. Panels (**a**,** d**) represent samples immediately after fermentation (FOB and FMB); (**b**,** e**) after simulated gastric digestion (FOB-G and FMB-G); and (**c**,** f**) after simulated intestinal digestion (FOB-I and FMB-I). Cells were stained with Syto-9 (live cells) and propidium iodide (PI, dead cells). Quadrants represent Q1 (dead cells), Q2 (injured cells), Q3 (live cells), and Q4 (unstained cells or beverage components). Panels are representative of triplicate analyses

To deliver a more nuanced assessment, flow cytometry dot plots (Fig. 2) allowed the differentiation of three cellular states: live cells (Syto9 + and PI-), injured cells (Syto9 + and PI+), and dead cells (Syto9- and PI+).

**Fig. 3:**
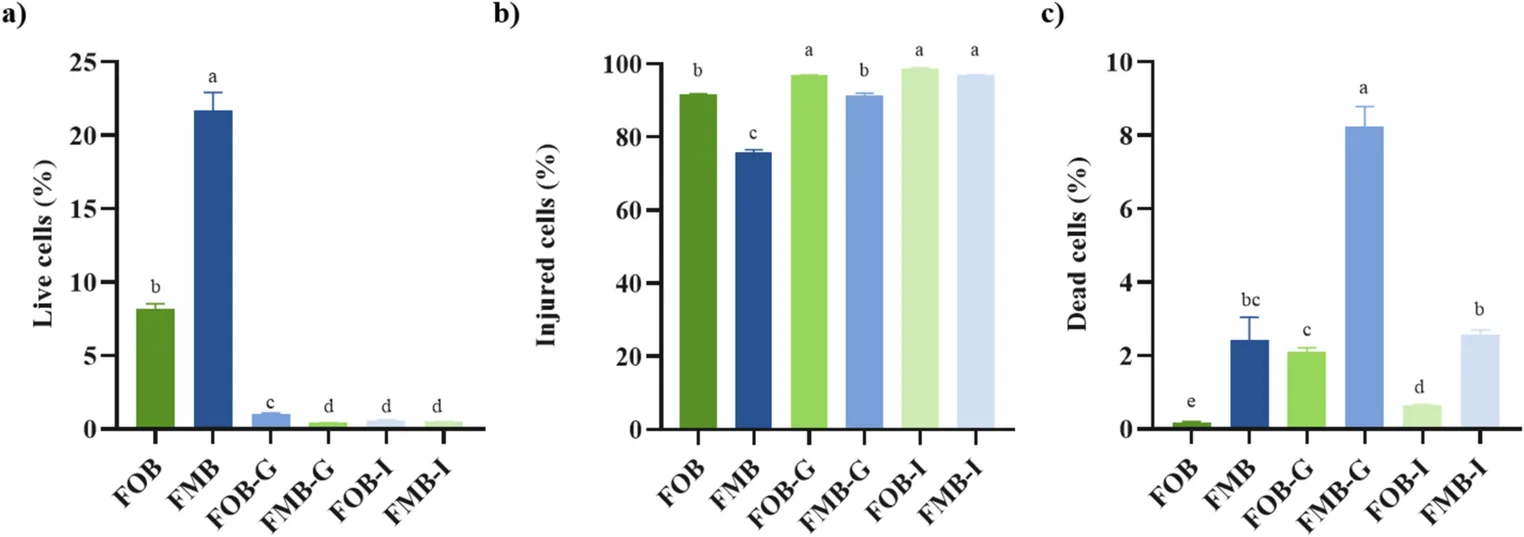
Cellular physiological state of *L. rhamnosus* LRB in fermented oat beverage (FOB) and fermented milk beverage (FMB) before and during simulated in vitro digestion: (**a**) live cells, (**b**) injured cells, and (**c**) dead cells. FOB and FMB correspond to samples immediately after fermentation; FOB-G and FMB-G to the gastric phase; and FOB-I and FMB-I to the intestinal phase. Different letters indicate significant differences among all treatment combinations across both beverages and digestion stages simultaneously within each physiological state (**a**, **b**, and **c**) independently, according to Tukey’s multiple comparisons test (*p* < 0.05)

The FOB initially had a lower proportion of intact membrane (live) than the FMB (8.20% ± 0.33 and 21.67% ± 1.25, respectively, *p* < 0.001; Figs. 2 and 3). Notably, despite the lower initial viability, the FOB-G demonstrated superior resilience under simulated gastric conditions, retaining a significantly higher percentage of live cells (1.02% ± 0.08) compared to the FMB-G (0.41% ± 0.02; *p* = 0.02). This matrix-mediated protection is reinforced by the significantly lower percentage of dead cells in the FOB-G (2.10% ± 0.11) compared to the FMB-G (8.23% ± 0.55, *p* < 0.001), a protective advantage that persisted through the intestinal phase (0.63% ± 0.03 versus 2.58% ± 0.13, respectively).

Rather than a mere substrate effect, the protection observed is probably driven by a multi-targeted mechanism characteristic of oat matrices. Structurally, the interaction between oat $\:\beta\:$-glucans and added inulin forms a dense, high-viscosity cross-linked hydrogel [27]. This microenvironment acts as a physical barrier that may restrict the diffusion of aggressive gastric acids and hydrophobic bile salts, thereby delaying cell exposure and subsequent rupture of the bacterial membrane [28]. Furthermore, the chemical composition of oats—rich in polyphenols, flavonoids, and active carboxylic acid derivatives—may provide an antioxidant buffer that neutralizes oxidative stress-induced cellular damage during digestive transit [29, 30]. Metabolically, the rapid initial pH drop during FOB fermentation serves as an environmental cue that triggers acid-adaptation mechanisms, such as exopolysaccharide (EPS) synthesis and stress-protein expression by *L. rhamnosus* LRB [27]. These structural and physiological alterations confer cross-protection to the cells, supposedly explaining the markedly lower cell death rate observed in the oat matrix compared to the dairy control, where delayed acidification minimized these adaptive responses.

Injured cells were the predominant population in all digestive stages (> 90%). This cell damage is likely due to structural stress and the acidic conditions encountered during the gastric phase, which increase membrane permeability, resulting in double-positive labeling (PI and Syto-9) indicating intact and permeable membranes [31]. Interestingly, these high percentages of injured cells contrast with the high counts obtained via the plate method (Fig. 1), suggesting that many cells in the “injured” state retain the ability to recover and form colonies under favorable culture conditions. This highlights that FC offers a more sensitive and nuanced assessment of the physiological state, detecting viable but non-culturable (VBNC) cells that complement traditional measurements.

### Cellular Morphology of the *L. rhamnosus* LRB after *InVitro* simulated digestion

**Fig. 4:**
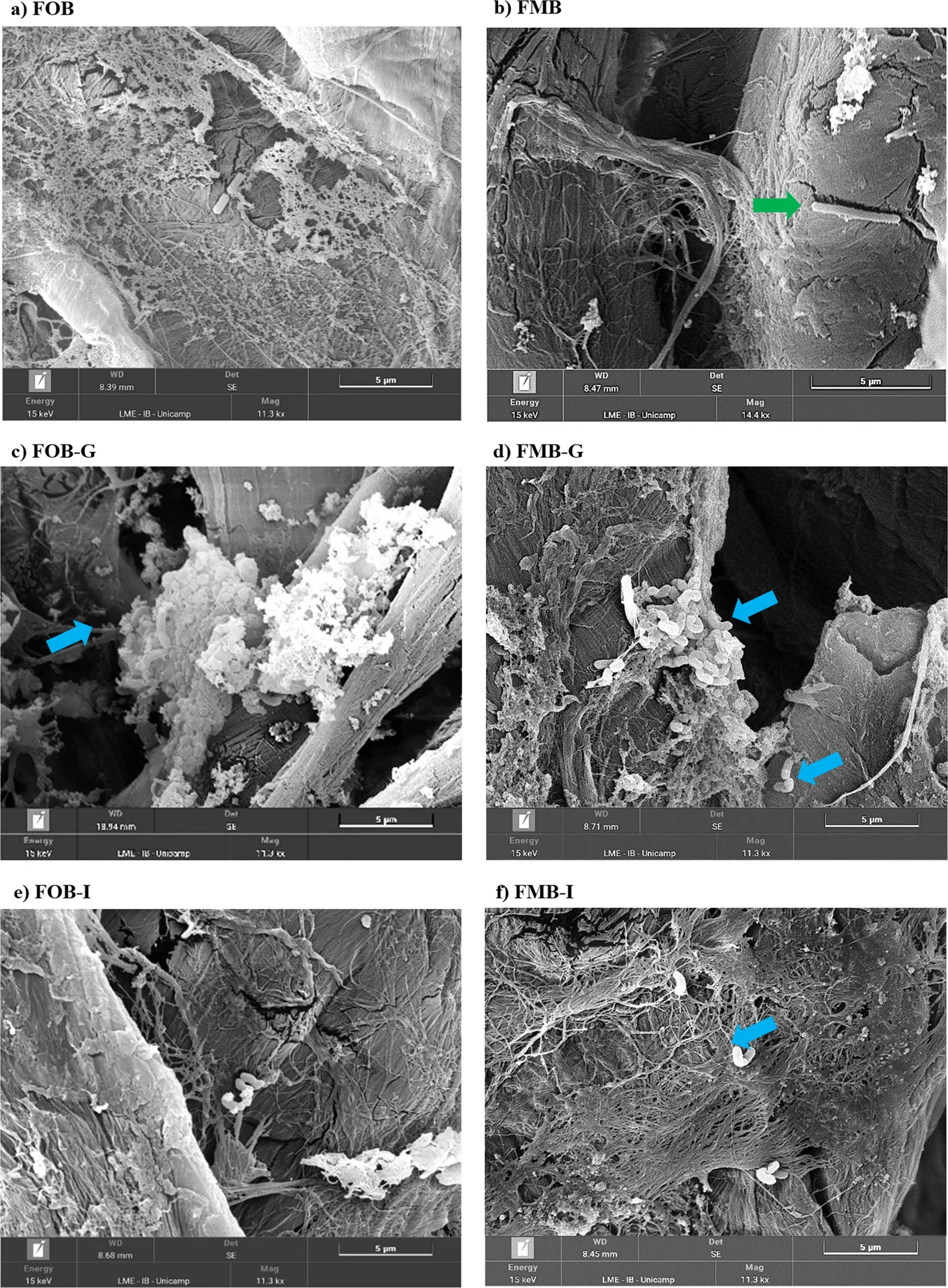
Scanning electron microscopy of *L. rhamnosus* LRB in fermented oat beverage (FOB) and fermented milk beverage (FMB) before and during simulated in vitro digestion. Panels (**a**, **c**, **e**) correspond to FOB and (**b**, **d**, **f**) to FMB. Panels (**a**, **b**) represent samples immediately after fermentation (FOB and FMB); (**c**, **d**) after simulated gastric digestion (FOB-G and FMB-G); and (**e**, **f**) after simulated intestinal digestion (FOB-I and FMB-I). Arrows indicate cell multiplication (green), and curved rod morphology (blue). Magnification: 10,000–15,000x

Images of each stage of digestion were obtained by scanning electron microscopy (SEM) to evaluate bacterial morphology and the protective roles of the plant and animal matrices (Fig. 4). Additionally, complementary SEM images for each sample were provided in Figure S1 (Supplementary Material) to ensure a more representative visual assessment of the treatments. The micrographs reveal the lactic culture cells, the food microstructure at different stages, and the polyether sulfone membrane network (used as a support for microorganism collection). 

Prior to digestion, *L. rhamnosus* LRB cells exhibited intact cell walls and typical rod-shaped profiles in both matrices (Fig. 4a, b). Cell division was evident in the FMB (green arrow, Fig. 4b). Structural damage was observed after digestion. Gastric acid stress induced curved bacillary shape (blue arrow, Fig. 4c and d), which correlates with the high proportion of injured cells detected via FC. These irregular and curved morphologies likely reflect structural stress on the bacterial cell envelope induced by the harsh environment. Lactic acid bacteria characteristically respond to these stresses and to acidity through complex cell wall remodeling, stress protein production, and alterations in membrane composition to maintain homeostatic integrity [32, 33].

During the intestinal phase, a similar pattern of cell fragmentation, surface roughness, and irregular morphology was observed (Fig. 4e and f). Comparable structural alterations, such as folding, thinning, and membrane roughness, have been reported in cells exposed to bile acids [34]. Concomitantly, disruption of the milk protein network and other matrix components was observed, consistent with findings reported for dairy matrices [12]. These structural modifications reflect the adaptive phenotypic responses and intrinsic pathways—such as exopolysaccharide production or cell-envelope remodeling—triggered by the strain to resist environmental challenges [35]. Although gene expression analysis was outside the scope of this work, the morphological and cytometric evidence provided here provides clear insights into matrix-mediated shielding mechanisms, establishing a robust basis for high-performance plant-based synbiotic products. Nevertheless, it must be emphasized that the observed structural alterations and survival rates stem from a static in vitro model, which has inherent limitations and cannot replicate dynamic peristalsis, continuous enzymatic secretions, or the complex interplay with the native microbiota of the human gastrointestinal tract.

## Conclusions

This study demonstrates that the developed oat beverage serves as a highly responsive, non-dairy vehicle for *L. rhamnosus* LRB, outperforming the traditional bovine milk matrix in terms of acidification. Rather than acting as a substrate, the oat-inulin matrix promotes a functional microenvironment that triggers acid-stress adaptation and provides structural shielding, thereby mitigating environmental challenges during refrigerated storage and gastrointestinal transit. The physiological resilience—evidenced by the maintenance of high culturability alongside viable but non-culturable cells—combined with the preservation of residual antioxidant properties, underscores the synbiotic potential of this formulation. These findings provide valuable insights for the food industry, demonstrating that plant-based matrices can achieve high performance and technical stability without relying on dairy components. Future research focusing on molecular adaptation pathways and in vivo validation will be the next essential steps to consolidate these achievements.

## Supplementary Information

Below is the link to the electronic supplementary material.

Supplementary Material 1 (PDF 939 KB)

## Data Availability

No datasets were generated or analysed during the current study.
