# Supplementary material for: Survival of Lacticaseibacillus rhamnosus LRB in a Potential Synbiotic Oat Beverage: Insights into Cell-Matrix Interactions During In Vitro Digestion
Source: Plant Foods Hum Nutr. 2026 Jul 22;81(3):99. doi: 10.1007/s11130-026-01550-9 (PMC13391689; doi:10.1007/s11130-026-01550-9)
Supplement: Supplementary file 1 — Supplementary Material 1 (PDF 939 KB) [file 11130_2026_1550_MOESM1_ESM.pdf]

Survival of *Lacticaseibacillus rhamnosus* LRB in a potential synbiotic oat beverage: Insights into cell-matrix interactions during *in vitro* digestion

Giovanna Alexandre Fabiano[a], Douglas Xavier dos Santos[b], Stefhani Andrioli Romero[a], Rodrigo Stein Pizani[a], Paulo César Martins Alves[c], Marcos Tadeu Nolasco da Silva[c], Mauricio Ariel Rostagno[a], Adriane Elisabete Costa Antunes[a*].

[a]School of Applied Sciences, Universidade Estadual de Campinas (UNICAMP), Limeira, SP, Brazil; [b]Department of Food Science and Nutrition, Faculty of Food Engineering, Universidade Estadual de Campinas (UNICAMP), Campinas, SP, Brazil; [c]Center for Investigation in Pediatrics, School of Medical Sciences, Universidade Estadual de Campinas (UNICAMP), Campinas, SP, Brazil. *Corresponding author: adriane@unicamp.br

Supplementary Material

1. Details of the materials and methods section

Preparation and fermentation of beverages

The titratable acidity was calculated according to Equation (S1)[1]:

$$Lactic\ acid\ (\%, w/w) = \frac{V_{NaOH} \times M \times f \times 90}{W\ sample \times 10} \quad \text{(Eq. S1)}$$

Where V_{NaOH} is the volume of NaOH solution used (mL), M is the molarity of the NaOH solution (0.1 mol/L), f is the correction factor from standardization, 90 is the molar mass of lactic acid (g/mol), $W\ sample$ is the sample weight (g), and 10 is the factor to express the result as a percentage.

Characterization of the fermented oat beverage

All proximate composition parameters were determined in triplicate according to the official methods of the Association of Official Analytical Chemists[1].

Moisture content was determined by the oven-drying method. Aliquots of the beverages (2.0 g) were weighed into previously dried and tared Petri dishes. The samples were dried in a forced-air oven at 100–105 °C for 24 h. After cooling in a silica gel

desiccator, the plates were reweighed, and moisture percentage was calculated by weight loss using Eq. S2:

$$Moisture\ (\%) = \frac{Initial\ weight\ (g) - Final\ weight\ (g)}{Initial\ weight\ (g)} X\ 100\ \text{(Eq. S2)}$$

Ash content was determined by incineration using a muffle furnace. Approximately 5.0 g of each sample was weighed into previously ignited and tared crucibles. The samples were incinerated at 500–550 °C for up to 24 h until a white, homogeneous ash was obtained. The ash percentage was calculated using Eq. S3:

$$Ash\ (\%) = \frac{Ash\ weight\ (g)}{Sample\ weight\ (g)} X\ 100\ \text{(Eq. S3)}$$

Total protein content was quantified by the Kjeldahl method to determine total nitrogen. Samples were digested with concentrated sulfuric acid (H_2SO_4) in the presence of a catalyst mixture, followed by steam distillation. The distillate was titrated with standardized hydrochloric acid (HCl). Total nitrogen (% TN) and total protein (% P) contents were calculated using Eq. S4 and S5, respectively:

$$TN\ (\%) = \frac{V\ X\ N\ X\ 14\ X\ 100}{P}\quad \text{(Eq. S4)}$$

$$P\ (\%) = \%\ \text{TN X}\ Fc\quad \text{(Eq. S5)}$$

Where *V* is the volume of HCl used in titration (mL), *N* is the normality of HCl, *P* is the sample weight (g), and *Fc* is the specific nitrogen conversion factor, established as 5.75 for plant-proteins[2].

Lipid content was determined by continuous extraction using a Soxhlet apparatus. Aliquots of the beverages were transferred to filter paper cartridges and extracted using petroleum ether as the solvent. After the extraction period, the solvent was evaporated, and the flasks were dried to constant weight. The lipid percentage was calculated using Eq. S6:

$$Lipids\ (\%) = \frac{Final\ flask\ weight\ (g) - Initial\ flash\ weight\ (g)}{Sample\ weight\ (g)}\ X\ 100\ \ \text{(Eq. S6)}$$

Total carbohydrates were determined by difference, subtracting the sum of moisture, ash, protein, and lipid percentages from 100%, according to Eq. S7:

$$Total\ carbohydrates\ (\%) = 100 - (M + A + P + L) \quad \text{(Eq. S7)}$$

Where M represents the total percentage of moisture, A is the ash, P is the protein, and L is the lipids.

Microbiological quality was evaluated at the end of the 28-day refrigerated storage period, in accordance with Brazilian regulatory standards (Normative Instruction No. 161 of July 1, 2022) for refrigerated processed beverages[3]. For all analyses, 25 mL of each sample was homogenized in 225 mL of sterile saline solution (0.9% NaCl), followed by serial decimal dilutions (10^{-3}) in sterile saline.

Presumptive *Bacillus cereus* was enumerated according to the American Public Health Association (APHA) protocol[4]. Aliquots (0.1 mL) of the serial dilutions were plated into Mannitol Egg Yolk Polymyxin (MYP) agar. The plates were incubated at 30–32 °C for 24 h. Typical colonies were counted, and results were expressed as colony-forming units per milliliter (cfu.mL^{-1}).

Enterobacteriaceae were enumerated using the pour-plate method with Violet Red Bile Glucose (VRBG) agar, following APHA guidelines[5]. After pour-plating 1.0 mL of the serial dilutions and complete solidification of the medium, an overlay of the same agar was added. The plates were incubated at 35 °C for 18–24 h. Typical colonies were counted, and results were expressed as cfu.mL^{-1}.

Molds and yeasts were enumerated using the surface-plating method on Sabouraud Agar according to APHA protocols[6]. Aliquots (0.1 mL) of the serial dilutions were inoculated and incubated at 25 °C for 5 days. Typical mold and presumptive yeast colonies were quantified, and results were expressed as cfu.mL^{-1}.

The presence or absence of *Salmonella* spp. was determined according to the Bacteriological Analytical Manual of the Food and Drug Administration (FDA/BAM) protocol[7]. For pre-enrichment, a 25 mL aliquot of each beverage was homogenized in 225 mL of Lactose Broth and incubated at 35 °C for 24 h. Subsequently, selective enrichment was performed by transferring the pre-enriched culture into Tetrathionate (TT) Broth and Selenite Cystine (SC) Broth, followed by incubation at 35 °C for 24 h. For differential

plating, loops of the selective broths were streaked onto Hektoen Enteric (HE) agar, Xylose Lysine Deoxycholate (XLD) agar, and Salmonella-Shigella (SS) agar, and incubated at 35 °C for 24 h to observe typical colonies. Presumptive colonies were provisionally confirmed using Lysine Iron Agar (LIA) and Triple Sugar Iron (TSI) agar slants (35 °C, 24 h). Final serological identification of suspicious colonies was carried out using a polyvalent somatic and flagellar antiserum kit (Probac, Brazil). Results were expressed as presence or absence of *Salmonella* spp. in 25 mL.

Antioxidant activity assays

All antioxidant activity assays were performed in microplates. The 2,2-diphenyl-1-picrylhydrazyl (DPPH) radical scavenging activity was conducted using a 150 µM DPPH solution in ethanol, prepared by diluting a 500 µM stock solution (10.9 mg of DPPH in 50 mL of absolute ethanol)[8]. A Trolox calibration curve was constructed with standard concentrations ranging from 20 to 140 µM. Aliquots of 66 µL of each sample, standard, blank (sample solvent), or control (absolute ethanol) were pipetted into the microplate wells. The mixture was allowed to react in the dark for 45 min, and the absorbance was measured at 517 nm.

The Trolox equivalent antioxidant capacity (ABTS) was evaluated by preparing an ABTS•+ radical cation solution reacting 7 mM ABTS stock solution with 140 mM persulfate in potassium phosphate buffer (75 mM, pH 7.4)[9]. The mixture was allowed to react in the dark for 16 h at room temperature. Before, the ABTS solution was diluted with the buffer to an absorbance of 0.70 ± 0.02. A Trolox calibration curve was prepared using analytical points ranging from 12.5 to 200 µM. Aliquots of 20 µL of each sample or standard were pipetted into the microplate wells, followed by the addition of 220 µL of the adjusted ABTS radical solution. Phosphate buffer (240 µL) was used as the blank. The microplate was incubated in the dark for 6 min, and the absorbance was measured at 730 nm.

The Ferric Reducing Antioxidant Power (FRAP) was evaluated using a FRAP reagent freshly prepared before each analysis by mixing a 0.3 M potassium acetate buffer solution (pH 3.6), a 10 mM 2,4,6-tris(2-pyridyl)-s-triazine (TPTZ) solution (solubilized in 40 mM HCl), and a 20 mM ferric chloride (FeC) aqueous solution in a 10:1:1 (v/v/v) ratio[10]. For the calibration curve, a 2500 µM ferrous sulfate ($FeSO_4$) aqueous stock

solution was prepared and appropriately diluted to establish the standard concentrations. Aliquots of 20 µL of each sample, standard, or distilled water (blank) were transferred to the microplate wells, followed by the addition of 30 µL of distilled water and 200 µL of the FRAP reagent. The microplate was shaken and incubated at 37 °C for 8 min. The absorbance was measured at 595 nm. Antioxidant activity was quantified using the calibration curve of $FeSO_4$, plotting concentration on the y-axis against absorbance on the x-axis, and results were expressed as ferrous sulfate equivalents.

***Storage stability and viability of L. rhamnosus* LRB**

The survival of *L. rhamnosus* LRB during storage was evaluated by the logarithmic variation (Δ log) of the counts, calculated according to Equation (S8)[11]:

$$\Delta\, log\ = log_{10}\,(N)\ - log_{10}\,(N_0) \quad \text{(S8)}$$

Where N represents the final population, and N_0 is the initial population immediately after fermentation.

2. Details of the results and discussion section

Characterization of fermented oat beverage

The oat beverage fermented by *L. rhamnosus* LRB was characterized regarding its proximate composition (Table S1), microbiological safety (Table S2), and antioxidant activity (Table 3).

Table S1. Proximate composition (%) of oat beverage fermented by *L. rhamnosus* LRB.

	Moisture	Ashes	Proteins	Lipids	Carbohydrates
**Fermented oat beverage**	89.34 ± 0.13	0.21 ± 0.08	0.64 ± 0.19	0.69 ± 0.06	9.12 ± 0.44

Values described as mean ± standard deviation.

Table S2. Microbiological quality of the oat beverage fermented by *L. rhamnosus* LRB during the refrigerated storage (5 - 10 °C).

**Storage time (days)**	*B. cereus*	Enterobacteriacea	Molds and yeasts	*Salmonella* ssp.
**0**	<10	<10	<10	Absent
**28**	<10	<10	<10	Absent

Values described as mean ± standard deviation. cfu ± /mL for *B. cereus*, Enterobacteriacea, molds and yeasts, and 25mL for *Samonella* ssp.

Cellular morphology of the L. rhamnosus LRB after in vitro simulated digestion

Additional images obtained by scanning electron microscopy (SEM) of the bacterial morphology of *L. rhamnosus* LRB in fermented oat beverage and fermented milk beverage (Figure S1).

a) FOB

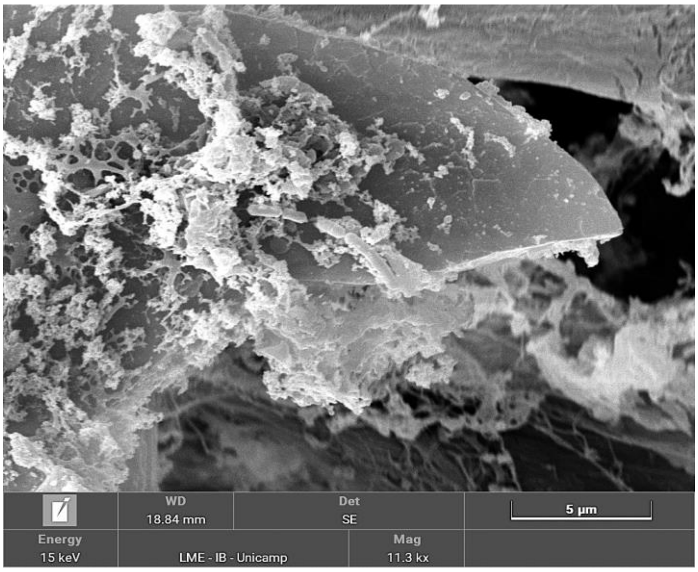

b) FMB

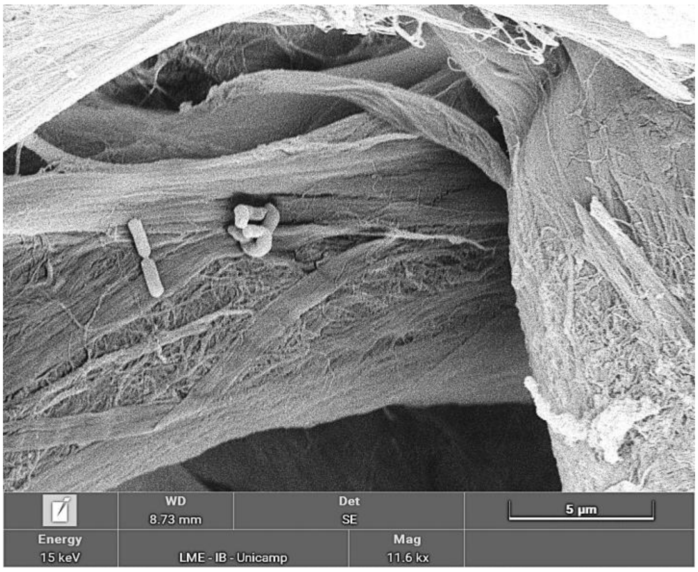

c) FOB-G

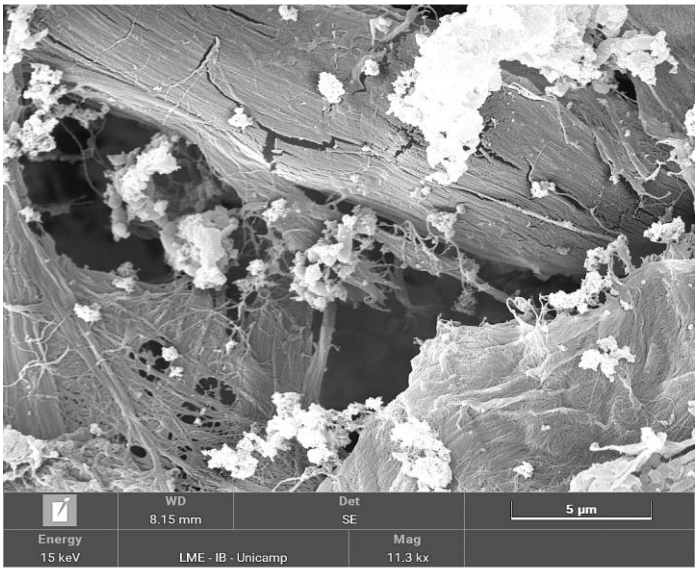

d) FMB-G

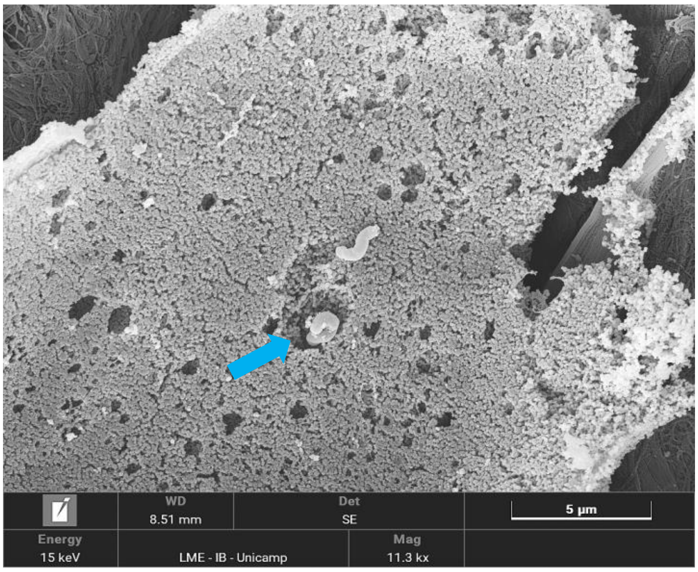

e) FOB-I

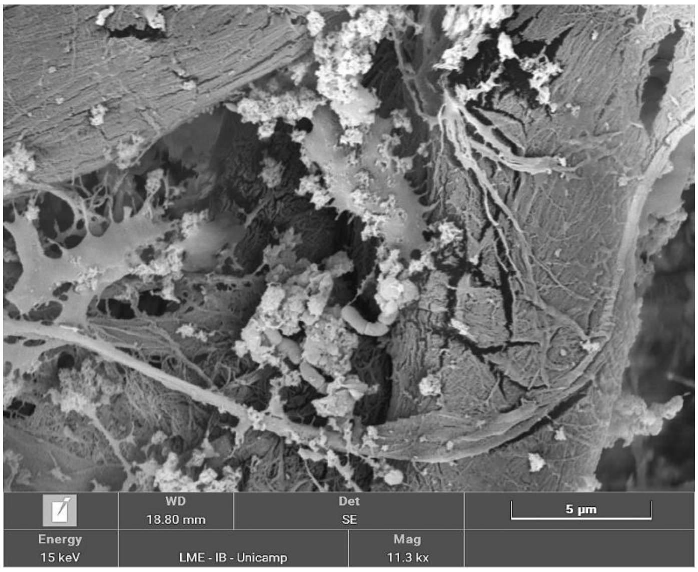

f) FMB-I

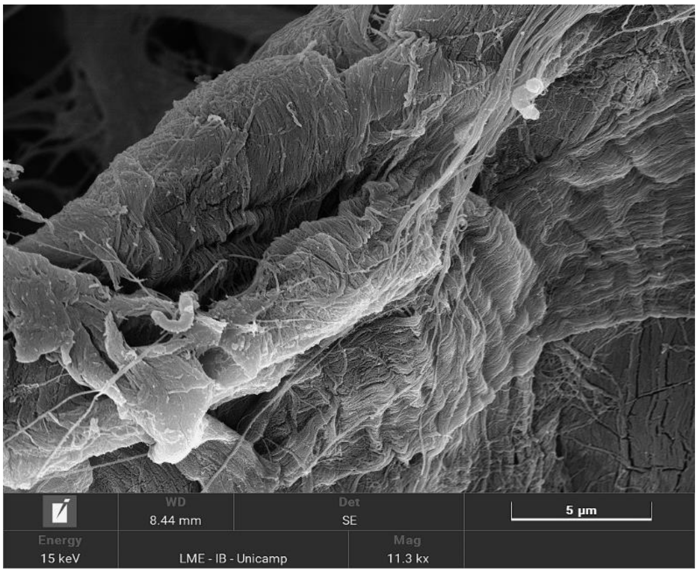

Fig S1. Additional imagens for scanning electron microscopy of *L. rhamnosus* LRB in fermented oat beverage (FOB) and fermented milk beverage (FMB) before and during simulated *in vitro* digestion. Panels **(a, c, e)** correspond to FOB and **(b, d, f)** to FMB. Panels **(a, b)** represent samples immediately after fermentation (FOB and FMB); **(c, d)** after simulated gastric digestion (FOB-G and FMB-G); and **(e, f)** after simulated intestinal digestion (FOB-I and FMB-I). Arrows indicate curved rod morphology (blue). Magnification: 10,000–15,000×.

References

1. AOAC. (2005). Official Methods of Analysis of AOAC International (18th ed.). Gaithersburg, MD, USA: AOAC International.
2. Brasil. Ministério da Saúde. Agência Nacional de Vigilância Sanitária (2003) Resolução - RDC Nº360, de 23 de dezembro de 2003. Brasília, Brazil. Avaliable at: https://anvisalegis.datalegis.net/action/ActionDatalegis.php?acao=abrirTextoAto&tipo=RDC&numeroAto=00000360&seqAto=000&valorAno=2003&orgao=RDC/DC/ANVISA/MS&codTipo=&desItem=&desItemFim=&cod_menu=1696&cod_modulo=134&pesquisa=true. Accessed 30 Nov 2023.
3. Brasil. Ministério da Saúde. Agência Nacional de Vigilância Sanitária (2022) Normative Instruction No. 161, 1 July 2022. Brasília, Brazil. Available at: https://antigo.anvisa.gov.br/documents/10181/2718376/IN_161_2022_.pdf/b08d70cb-add6-47e3-a5d3-fa317c2d54b2. Accessed 30 Nov 2023.
4. Bennet R, Tallent S, Hait J (2015) *Bacillus cereus* and *Bacillus cereus* toxins. In: Salfinger Y, Tortorello ML (Eds.), Compendium of methods for the microbiological examination of foods (5th ed., pp. 385-397). Washington, DC, USA: American Public Health Association (APHA).
5. Kornacki J, Gurtler J, Stawick B (2015) Enterobacteriaceae, Coliforms, and *Escherichia col*i as quality and safety indicators. In: Salfinger Y, Tortorello ML (Eds.), Compendium of methods for the microbiological examination of foods (5th ed., pp. 411-428). Washington, DC, USA: American Public Health Association (APHA).
6. Ryu D, Wolf-Hall C (2015) Yeasts and molds. In: Salfinger Y, Tortorello ML (Eds.), Compendium of methods for the microbiological examination of foods (5th ed., pp. 429-439). Washington, DC, USA: American Public Health Association (APHA).
7. Andrews W, Wang H, Jacobson A, Ge B, Zhang G, Hammack T (2007) BAM: *Salmonella*. In: Bacteriological Analytical Manual (BAM). U.S. Food and Drug Administration (FDA), College Park, MD, USA.
8. Brand-Williams W, Cuvelier ME, Berset C (1995) Use of a free radical method to evaluate antioxidant activity. LWT 28:25–30 http://doi.org/10.1016/S0023-6438(95)80008-5
9. Van Den Berg R, Haenen GRMM, Van Den Berg H, Bast A (1999) Applicability of an improved Trolox equivalent antioxidant capacity (TEAC) assay for evaluation of antioxidant capacity measurements of mixtures. Food Chem 66:511–517 http://doi.org/10.1016/S0308-8146(99)00089-8
10. Benzie IFF, Strain JJ (1996) The Ferric Reducing Ability of Plasma (FRAP) as a measure of “antioxidant power”: the FRAP assay. Anal Biochem 239:70–76 http://doi.org/10.1006/abio.1996.0292
11. Peleg M, Corradini MG (2011) Microbial growth curves: what the models tell us and what they cannot. Crit Rev Food Sci Nutr 51:917–945 https://doi.org/10.1080/10408398.2011.570463
